# Problematic Digital Media Use and Behavioral Issues in Children with Special Needs: A Family Needs-Centered Perspective †

**DOI:** 10.3390/bs15111478

**Published:** 2025-10-30

**Authors:** Hülya Torun Yeterge

**Affiliations:** Department of Special Education, Faculty of Education, Adıyaman University, Adıyaman 02040, Türkiye; htorunyeterge@adiyaman.edu.tr

**Keywords:** problematic digital media use, behavioral problems, family needs, children with special needs, autism spectrum disorder (ASD)

## Abstract

As technological developments proliferate, understanding the impact of digital media on children with special needs has become increasingly critical. This study examines problematic digital media use, behavioral problems, and family needs among children with special needs, and investigates whether these variables differ by disability type, parental education, and socioeconomic status (SES). Parents of 357 children with special needs from various regions of Türkiye participated. Data were collected via parent-report questionnaires and analyzed using descriptive statistical techniques. Findings indicate high levels of problematic media use, behavioral problems, and family needs in this population. In particular, children with autism spectrum disorder (ASD) exhibited higher levels of problematic media use and behavioral problems, and their parents reported greater support needs than other groups. Conversely, lower levels of problematic media use and behavioral problems were observed among children whose parents had higher education and SES. Significant positive correlations also emerged among problematic media use, behavioral problems, and family needs. These findings suggest that appropriately guided digital media use may be associated with fewer behavioral difficulties and that strengthening supports for families could be a relevant target for intervention; however, causal inferences are not warranted from these data.

## 1. Introduction

The contemporary digital age has brought profound changes to access to information and human communication. By facilitating the ways in which information is obtained, the digital era enables faster and more comprehensive flows of information for all individuals. Modes of communication have shifted from face-to-face interactions to online platforms under the influence of digital tools, thereby strengthening global connectedness (Misra, 2023; Sahmal et al., 2025). As a result, the dynamics of social interaction and sociability have been fundamentally transformed. While this transformation yields benefits such as rapid communication and enhanced engagement, it has also been accompanied by the emergence of cyberbullying, abuse, and various problem behaviors (Armaou et al., 2024; Paltacı, 2024). With digitalization, individuals’ needs have diversified, and behaviors have taken new forms (Batur, 2024). This process affects all segments of society and exerts particularly pronounced effects on children with special needs and their families. Individuals with special needs are especially vulnerable to risks in the digital world, which in turn heightens families’ support needs (Wahyuningrum et al., 2020). Accordingly, understanding the implications of these digital-era changes for individuals with special needs is critically important for both scholarship and policy development (Rice & Cun, 2021; Wyeth et al., 2023).

Families of individuals with special needs face increasing and diversifying requirements in the domains of social support, lifelong learning, and digital parenting (Altındağ Kumaş & Sardohan Yildirim, 2024; Bjørlykhaug et al., 2022). In addition to financial and social support needs, families’ educational needs have become more complex amid an evolving digital landscape (Altındağ Kumaş & Sardohan Yildirim, 2024; Vossen et al., 2024; Zhong et al., 2020). Digital parenting, in particular, has emerged as a salient demand that complicates parents’ efforts to guide their children in a period of rapid technological change. Families who lack knowledge and skills in this area often struggle to contribute effectively to their children’s development (Papadopoulos, 2021). When needs are insufficiently met, families experience significant constraints in supporting the social and academic success of their children with special needs (Yelseli & Şener, 2021). This situation lowers family quality of life and may hinder children from reaching their potential. Family quality of life also varies by the child’s disability type and severity; indeed, families of children with more severe disabilities have been reported to experience lower quality of life (Çalbayram, 2024; Law et al., 2014). In light of the heightened demands associated with greater disability severity, developing comprehensive social support programs to address these needs is critical for both families and children. Accordingly, further research and policy development targeting parents—particularly in digital parenting and lifelong learning—are warranted (Altındağ Kumaş & Sardohan Yildirim, 2024; Bjørlykhaug et al., 2022; Zhong et al., 2020; Papadopoulos, 2021).

There appears to be a possible association between families’ unmet educational, financial, and social support needs, their daily fatigue, and a greater allowance of screen time for their child with special needs (Bozoğlan & Kumar, 2022; Caicedo, 2014; Mazurek & Wenstrup, 2013; Öztürk & Alemdar, 2023). This pattern may increase the likelihood of problematic digital media use among children with special needs (Craig et al., 2021; Rega et al., 2023). Problematic digital media use is characterized by excessive or uncontrolled engagement with digital media devices and is typically associated with addiction-like behaviors that adversely affect daily activities and academic and social functioning (Triantafyllou & Kapos, 2025). Families may mistakenly infer that their child exhibits fewer problem behaviors simply because the child appears quieter while using screens. Children with special needs who show higher levels of problematic digital media use may display more behavioral problems, possibly reflecting an association between increased screen exposure—both in terms of potentially harmful content and visual stimulation—and behavioral difficulties. Similarly, in typically developing young children, problematic screen use has been reported to be associated with autism-like symptoms (Craig et al., 2021; Ophir et al., 2023; Torun Yeterge, 2023; Westby, 2021).

Family needs play a pivotal role in childrearing and emerge as a critical factor in mitigating problematic behaviors among individuals with special needs (Eshraghi et al., 2022; Sikora et al., 2013). Meeting families’ social, psychological, and educational needs can enhance children’s behavioral adjustment and contribute to reductions in problematic behaviors (McWilliam, 2010; Upadhyay & Singh, 2024). In this context, developing effective family support programs is a fundamental requirement for improving the well-being of both children and their families (Christoforou et al., 2025). When families’ social, financial, and educational needs are addressed, they are better positioned to accurately identify their children’s needs and to spend higher-quality time with them, which may lower the likelihood of problematic digital media use. Among children with special needs, lower levels of problematic digital media use are often accompanied by fewer problem behaviors, thereby easing demands on families (Davis et al., 2023). Under such circumstances, improvements in children’s development and overall well-being may also be anticipated.

Meeting the needs of families of children with special needs is pivotal for facilitating non-problematic use of digital media tools and is likely associated with fewer problem behaviors. Effective use of digital media by families not only streamlines daily routines but may also contribute to reductions in children’s problem behaviors. Recent studies indicate that aligning digital media practices with family needs can help create developmentally supportive environments, suggesting that supporting family needs also supports the development of children with special needs (Bent et al., 2025; Fellin et al., 2024; Ying & Ai, 2025). Families should be supported across multiple domains and guided toward quality time outside the digital sphere. Evidence further suggests that meeting family needs can positively influence the development of children with special needs (Barklı & Doğan, 2025; Hernández, 2024). Accordingly, diversified supports that enable families to spend higher-quality time with their children and build awareness around digital media may foster more efficient media use and, in turn, be associated with fewer problem behaviors. Both the direct (e.g., guidance, skills training) and indirect (e.g., structural resources) needs of families regarding digital media should be addressed (Coşkunalp, 2022).

Addressing families’ needs is critically important in the context of problematic digital media use (Hernández, 2024). Access to adequate financial resources, educational opportunities, and social support enables parents of children with special needs to spend higher-quality time with their children (Güllüpınar, 2013; Kumcağız et al., 2018). This, in turn, provides a strong basis for shaping and regulating media-use habits for both parents and children. Research indicates that intra-family support mechanisms can help keep media use under control and are associated with fewer problem behaviors; moreover, children with special needs may be particularly responsive to guidance regarding digital media, with such guidance linked to improved behavioral adjustment (Bent et al., 2025; Hui et al., 2025). Conversely, limited social support and insufficient educational opportunities may constrain parents’ capacity to manage media use, potentially accompanying increases in media dependence and related problem behaviors. Accordingly, interventions for children with special needs and their families should examine, in a holistic manner, the interrelations among family needs, problematic digital media use, and problem behaviors.

The theoretical foundation of this study integrates the Family Stress Model and the Differential Susceptibility to Media Effects Model (DSMM). The Family Stress Model posits that lower socioeconomic status (SES) elevates parental stress, thereby constraining parenting practices and relating to children’s behavioral problems (Masarik & Conger, 2017). DSMM emphasizes that the effects of problematic digital media use are shaped by child characteristics (e.g., disability type) and family context (e.g., parental education/SES, parental mediation) (Kattein et al., 2023; Valkenburg & Peter, 2013). This framework informs the direction of our hypotheses (e.g., higher problematic use and behavioral difficulties under lower SES and lower parental education) and anticipates positive associations among the focal variables. Although prior research shows links between SES or education indicators and child/family outcomes, these associations do not inherently denote “better” family qualities; they more often reflect structural opportunities and constraints. Accordingly, our study frames SES/education-related differences not as normative superiority but as markers of unequal resource distribution. The study aims to identify the needs of families of children with disabilities and, by elucidating the interplay between problematic digital media use and behavioral difficulties, to inform strategies that may reduce behavioral challenges through more guided media practices. The existing literature on problematic digital media use and behavioral problems remains limited, and its intersection with family needs has been insufficiently addressed. Moreover, critical moderators—such as disability groups, parental education, and SES—are frequently overlooked. This study, therefore, seeks to help fill these gaps in the literature.

### 1.1. Research Aim and Questions

This study examines problematic digital media use among children with special needs, their behavior problems, and their families’ needs, and investigates whether these factors differ by children’s disability type, parents’ educational attainment, and socioeconomic status. In line with the study’s overarching aim, it addresses the following questions:

1. What are the levels of children’s problematic digital media use, behavior problems, and families’ needs?
2. Are there statistically significant associations among children’s problematic digital media use, behavior problems, and families’ needs?
3. Do children’s disability type, parental education level, and socioeconomic status differentiate levels of children’s problematic digital media use, behavior problems, and families’ needs?

### 1.2. Hypotheses

**H1.**  *Children with special needs will exhibit high levels of problematic digital media use.*

**H2.**  *Children with special needs will exhibit high levels of behavioral problems.*

**H3.**  *Parents of children with special needs in this study will report high levels of family needs as measured by the Family Needs Assessment Tool (FNAT).*

**H4.**  *There is a positive association between problematic digital media use and behavioral problems.*

**H5.**  *There is a positive association between problematic digital media use and family needs.*

**H6.**  *There is a positive association between family needs and behavioral problems.*

**H7.**  *Disability type will be associated with differences in children’s problematic digital media use, behavioral problems, and family needs (note: effect sizes may be limited for categories with small cell sizes).*

**H8.**  *Parental education level will be associated with differences in children’s problematic digital media use, behavioral problems, and family needs (note: effect sizes may be limited for categories with small cell sizes).*

**H9.**  *Socioeconomic status (SES) will be associated with differences in children’s problematic digital media use, behavioral problems, and family needs (note: effect sizes may be limited for categories with small cell sizes).*

## 2. Research Model

This study employed a correlational survey design, and data were collected between August and December 2024. Correlational designs aim to determine the presence and magnitude of associations between two or more variables (Karasar, 2012). Accordingly, the model examined differences and associations among three independent variables—(i) child disability type, (ii) parental education level, and (iii) family socioeconomic status—and three key dependent variables concerning children with special needs and their families: (a) child behavior problems, (b) problematic digital media use, and (c) family needs.

### 2.1. Participants

A total of 357 parents were included, meeting the inclusion criteria of residing in various regions of Türkiye, having a child with special needs, and volunteering to participate. Each parent reported on one child (N = 357). Of the participating parents, 216 were mothers and 141 were fathers. Children’s disability categories were as follows: intellectual disability (ID) n = 96, autism spectrum disorder (ASD) n = 162, multiple disability (MD) n = 31 (defined as ASD plus a physical disability, or intellectual plus physical disability), visual impairment (VI) n = 16, hearing impairment (HI) n = 20, and other n = 32 (learning disability, physical disability, and/or attention-deficit/hyperactivity disorder).

Household socioeconomic status (SES), classified by monthly income, was distributed as very high (≥200,000 TL) 22%, high (50,000–200,000 TL) 23%, medium (20,000–50,000 TL) 34%, and low (0–20,000 TL) 21%. Regarding parental education, 20.5% were categorized as low, 37.5% as medium, and 42% as high (mutually exclusive categories summing to 100%). For analyses, parental education was operationalized as the mean of the two parents’ education levels: each parent’s attainment was coded numerically and averaged, then categorized as low (primary–middle school), medium (high school), or high (bachelor’s or graduate degree).

### 2.2. Data Collection

Data were collected on a voluntary basis after obtaining informed consent from all participants. All instruments and forms were prepared by the researcher and administered online. A combination of convenience and snowball sampling was used. Parents of children with special needs known to the researcher from 15 years of teaching experience were contacted by phone; for those who agreed to participate, the researcher read the questions and recorded responses in the online form. For some parents, paper forms were delivered and later transcribed by the researcher into an Excel file. The survey was implemented in three modes: researcher-administered telephone interview, paper form with subsequent transcription, and online self-report. For each participant, the administration mode was logged; mode-specific sample shares, item nonresponse rates, and completion practices were reported descriptively. Double data entry with reconciliation was used for paper forms; telephone interviews followed a standardized script and a neutral probing technique. The online mode emphasized privacy and time flexibility. The study did not aim to test quantitative differences across modes; rather, it documented potential mode-related bias transparently through process controls and descriptive indicators. Data were collected through multiple administration modes: 31% via telephone interviews, 35% through paper forms with transcription, and 34% through online self-report forms. Small differences in mean scores were observed across modes; however, these differences were reported descriptively only and were not statistically tested. Descriptive by-mode differences were small; no inferential by-mode tests were planned.

When parents had more than one child, responses were obtained for a single target child. Additional participants were recruited via referrals from enrolled families (snowballing), which improved accessibility. After checking for missing data, cases with missing values on any variable (less than 2% of the total data) were excluded using listwise deletion. The final analytic sample consisted of N = 357 participants, with no missing data in any of the analyses. Data collection took place between August and December 2024. Ethical approval was obtained from the Adıyaman University Social and Humanities Research and Publication Ethics Committee (Protocol No. 105; approval date: 25 July 2024), confirming that the study adhered to scientific and publication ethics.

### 2.3. Data Collection Tools

#### 2.3.1. Problematic Media Use Scale–Short Form (PMUS-SF)

Domoff et al. (2019) developed the Problematic Media Use Scale to assess problematic media use/screen addiction among children aged 4–11 years. In the present study, *digital media* refers to smartphones, tablets, computers/laptops, smart TVs, and game consoles, as well as content consumed on these devices (e.g., videos/streaming, online games), social media, and apps. Problematic digital media use is operationalized as difficulty controlling use, marked irritability when access is removed, family conflict surrounding media, and preoccupation with media (e.g., “Child becomes irritable when media is removed.”). The Turkish validation of the scale was conducted by Furuncu and Öztürk (2020). The short form comprises 9 items rated on a 5-point Likert scale (1 = never to 5 = always), yielding a total score range of 9–45, with higher scores indicating greater problematic use. Exploratory factor analysis supported a unidimensional structure explaining 57.6% of the variance, and confirmatory factor analysis indicated good model fit (χ^2^/df = 1.81, CFI = 0.99, RMSEA = 0.062, RMR = 0.041). In this study, the PMUS-SF was used to index children’s level of problematic digital media use.

#### 2.3.2. Revised Behavior Problem Checklist (RBPC)—Turkish Adaptation

Behavior problems were measured using the Turkish adaptation of the Revised Behavior Problem Checklist (RBPC; Quay & Peterson, 1996; Turkish adaptation: Kaner, 1999; Kaner & Uçak-Çiçekçi, 2000; Kaner & Büyüköztürk, 2008). Following confirmatory factor analysis, fit indices were within acceptable ranges (RMSEA = 0.085; RMR = 0.067; GFI = 0.70; AGFI = 0.68; NFI = 0.97; NNFI = 0.97; CFI = 0.97; IFI = 0.97). After item refinement, the Turkish form contains 62 items rated on a 3-point scale (0 = not a problem, 1 = moderate problem, 2 = severe problem), with a total score range of 0–124; higher scores indicate greater behavior problems. The RBPC comprises six subscales: (1) Attention Problems, (2) Conduct Disorder, (3) Socialized Aggression, (4) Anxiety/Withdrawal, (5) Psychotic Behavior, and (6) Motor Tension. The RBPC indexes six subscales, each defined operationally with illustrative behaviors reported by parents: Conduct Disorder: rule-breaking/noncompliance, argumentativeness, fighting, property damage, lying (disruptive, norm-violating patterns). Socialized Aggression: peer-context aggression/bullying or threats, group rule violations, truancy/roaming, inciting others (socially organized aggression). Attention Problems: distractibility, difficulties sustaining attention/focus, impulsivity, inability to remain seated. Anxiety/Withdrawal: excessive worry and fear, shyness/withdrawal, avoidance, somatic complaints reflecting anxious symptoms. Psychotic Behavior: developmentally incongruent odd/unusual thoughts or behaviors and apparent disturbances in reality testing (parent-report indicators; not for clinical diagnosis). Motor Tension: restlessness, fidgeting, tension, tightening/rigidity indicative of psychomotor agitation. In this study, the RBPC total and subscale scores were used to quantify children’s behavior problem levels.

#### 2.3.3. Family Needs Assessment Tool (FNAT)

Family needs were assessed using the *Aile Gereksinimlerini Belirleme Aracı* developed for use in Türkiye (Cavkaytar et al., 2014). The instrument includes 29 items rated on a 3-point Likert scale (1 = definitely no, 2 = unsure, 3 = definitely yes), yielding a total score range of 29–87; higher scores reflect greater perceived family needs. Items are organized into four domains: Financial Needs, Explaining the Child’s Condition to Others, Information Needs, and General/Community Services (e.g., “I need information about available community services.”). Exploratory factor analysis supported a four-factor solution explaining 49.62% of the variance. In this study, the FNAT total and domain scores were used to index families’ perceived needs.

### 2.4. Data Analysis

The SPSS 25 statistical package program was used for data analysis in this study. To determine which analyses were appropriate, skewness and kurtosis values were first calculated. These values ranged from −1.840 to −0.024. According to George and Mallery (2010), distributions can be considered approximately normal when skewness and kurtosis values fall between −2 and +2. Therefore, parametric tests were used in the analyses.

For all three measurement instruments, frequency, percentage, mean, and standard deviation values were calculated to identify the general levels of the variables. We operationalized ‘high’ as scores at/above the 75th percentile of our sample; therefore, H1–H3 refer to sample-relative elevation, not clinical cutoffs. In the second stage of analysis, the Pearson product–moment correlation coefficient was computed to examine the strength of the relationships between variables. Finally, one-way analysis of variance (ANOVA) was conducted to determine whether there were significant differences based on children’s disability types and parents’ educational and socioeconomic levels.

Before performing ANOVA, Levene’s test for homogeneity of variances was applied, and the results confirmed that the assumption of homogeneity was met. Therefore, the use of parametric analyses was deemed appropriate. However, as a non-parametric robustness check, the Kruskal–Wallis tests was also performed (see Table A1, Table A2, Table A3 and Table A4). The evaluation of effect sizes followed the thresholds proposed by Green and Salkind (2005): 0.01 (small), 0.06 (medium), and 0.14 (large).

### 2.5. Reflexivity and Bias Mitigation

Although the lead author’s 15 years of experience in special education facilitated recruitment, it also carries a risk of interpretive bias. To reduce this risk, analyses followed a pre-specified plan, all findings are fully reported, and coding/verification procedures were reviewed by an independent special education expert with interrater reliability checks. Any discrepancies were resolved by consensus.

## 3. Results

The findings are presented in parallel with the study’s research questions.

As shown in Table 1, internal consistency was very high for the PMUS-SF (α = 0.983). For the RBPC, subscale α coefficients ranged from 0.686 to 0.978; the Motor Tension subscale was marginally acceptable (α = 0.686), likely reflecting its small item count, whereas the remaining subscales were excellent (α ≥ 0.939). The RBPC total score showed excellent reliability (α = 0.993). For the Family Needs Assessment Tool (FNAT), α coefficients for subscales and the total score ranged from 0.952 to 0.994, indicating strong internal consistency across domains. On central tendency, the PMUS-SF mean indicated, on average, moderate levels of problematic media use with substantial variability across individuals (M = 3.19, SD = 1.28). The RBPC total score averaged 3.53, and the subscale pattern suggested that Conduct (M = 3.05), Socialized Aggression (M = 3.24), and Attention Problems (M = 3.11) were the most prominent parent-reported difficulties, whereas Anxiety/Withdrawal (M = 3.00), Psychotic Behavior (M = 2.80), and Motor Tension (M = 2.42) were comparatively lower. FNAT results showed the highest needs in General Support & Community Services (M = 3.28) and Need to Explain to Others (M = 3.11), with Informational Needs (M = 3.09) and Financial Needs (M = 3.05) also salient. The FNAT total (M = 2.87) indicates elevated overall family needs. The difference in α across the RBPC subscales (Psychotic Behavior α = 0.825; Motor Tension α = 0.686) is largely attributable to the number of items (approximately four and three items, respectively). Because Cronbach’s α is sensitive to scale length, shorter subscales tend to yield lower α coefficients even when item quality is acceptable. Taken together, the scale scores point to notable levels of problematic digital media use and behavior problems as reported by parents, alongside substantial family needs. While the cross-sectional design does not permit causal inference, the pattern suggests that addressing family needs may be associated with more manageable media use and behavior profiles and could be targeted through feasible supports. These results also imply that many parents may feel underprepared or under-resourced to guide and support their children in digital contexts.

Table 2 indicates pervasive and strong positive associations among the total scores and subscales of the PMUS-SF, RBPC, and FNAT. The correlation between PMUS-SF Total and RBPC Total is high (*r* = 0.812, *p* < 0.001); similarly, the association between PMUS-SF Total and FNAT Total is high (*r* = 0.827, *p* < 0.001). Correlations between PMUS-SF Total and the RBPC subscales range from 0.782 to 0.862, with the highest value for Psychotic Behavior (*r* = 0.862), followed by Motor Tension (*r* = 0.846). Correlations between PMUS-SF Total and the FNAT subscales range from 0.783 to 0.833; the strongest association is with Financial Needs (*r* = 0.833) and the weakest with General Support & Community Services (*r* = 0.783). The association between RBPC Total and FNAT Total is also significant and high (*r* = 0.798, *p* < 0.001), and correlations between RBPC Total and the FNAT subscales fall between 0.754 and 0.848, with the highest again for Financial Needs (*r* = 0.848). The very high inter-subscale correlations within instruments (FNAT ≈ 0.89–0.99; RBPC ≈ 0.95–0.99) reflect conceptual proximity among dimensions within each scale. Overall, these findings suggest that higher levels of problematic media use are associated with higher levels of children’s behavior problems and family needs, with financial needs and psychotic/motor tension dimensions appearing most central within this correlational pattern.

According to the Table 3 PMUS-SF results, the ASD group (M = 3.39) had a higher mean score than the other groups, and post hoc analyses indicated that individuals with ASD showed greater problematic media use compared to those with intellectual disability (ID); however, this difference was not statistically significant (*p* = 0.077, η^2^ = 0.03). In the RBPC results, the ASD group (M = 3.78) had the highest problem behavior score among all groups. Post hoc analyses revealed that the ASD group demonstrated significantly higher problem behavior scores than the ID, multiple disability (MD), and other disability (OTHER) groups (*p* = 0.001, η^2^ = 0.06). The FNAT results showed that the MD group (M = 3.49) had the highest mean family needs score, and this difference was statistically significant when compared to the ID group (*p* = 0.03, η^2^ = 0.03). Overall, these findings highlight that individuals with ASD tend to show higher levels of problematic media use and behavioral problems, whereas families of children with multiple disabilities report greater support needs.

Table 4 shows that PMUS-SF, RBPC, and FNAT scores differ significantly by parental socioeconomic status (SES). On the PMUS-SF, the low-SES group had a mean of 4.44 (on a 1–5 scale), whereas the very high-SES group had 1.36 (*F* = 702.80, *p* < 0.001, η^2^ = 0.86); post hoc comparisons followed the ordered pattern 1 > 2 > 3 > 4 (i.e., all adjacent contrasts significant). On the RBPC, group means were 4.85, 3.88, 2.83, and 2.52, respectively (*F* = 320.41, *p* < 0.001, η^2^ = 0.73), with all pairwise differences significant (1 > 2; 1 > 3; 1 > 4; 2 > 3; 2 > 4; 3 > 4). On the FNAT, means were 4.53, 3.97, 1.41, and 1.24 (*F* = 315.93, *p* < 0.001, η^2^ = 0.73); post hoc tests indicated 1 > 2; 1 > 3; 1 > 4; 2 > 3; 2 > 4; and 3 ≈ 4 (ns). Effect sizes were large (η^2^ ≈ 0.73–0.86), indicating robust group differences. Across all instruments, higher SES was associated with systematically lower levels of problematic media use, behavior problems, and family needs (Low > Medium > High > Very High).

Table 5 indicates significant and sizable differences across parental education groups on all three instruments (scores reported on their original 1–5 Likert metrics). For the PMUS-SF, group means were Low = 4.44, Medium = 3.80, High = 2.02; the omnibus test was significant, *F*(2, 354) = 304.49, *p* < 0.001, η^2^ = 0.63, and Tamhane/Dunnett-T3 post hocs confirmed the ordered pattern Low > Medium > High. For the RBPC, means likewise decreased with education (Low = 4.85; Medium = 3.85; High = 2.60), *F*(2, 354) = 530.39, *p* < 0.001, η^2^ = 0.75; all pairwise contrasts were significant (1 > 2; 1 > 3; 2 > 3). For the FNAT, means were Low = 4.53; Medium = 3.81; High = 1.22, *F*(2, 354) = 448.38, *p* < 0.001, η^2^ = 0.72; post hocs again yielded 1 > 2; 1 > 3; 2 > 3. Taken together, higher parental education is associated with systematically lower levels of children’s problematic digital media use and behavior problems, as well as lower reported family needs, with large effect sizes throughout. These patterns highlight the priority of targeted educational and social-support programs for families with lower educational attainment.

As shown in Table 6, Pearson product–moment correlation analysis was used to examine the relationships among the three main variables. The results indicated strong and statistically significant positive correlations among all variables (*p* < 0.001). Specifically, a high correlation was found between behavioral problems (RBPC) and problematic digital media use (PMUS-SF) (r = 0.812, 95% BCa CI [0.771, 0.845]), supporting Hypothesis 4. Similarly, behavioral problems (RBPC) were strongly correlated with family needs (FNAT) (r = 0.798, 95% BCa CI [0.744, 0.845]), supporting Hypothesis 5. In addition, problematic digital media use (PMUS-SF) showed a strong positive association with family needs (FNAT) (r = 0.827, 95% BCa CI [0.788, 0.859]), confirming Hypothesis 6. The positive direction of all correlations suggests that higher levels of problematic digital media use among children tend to be associated with increased behavioral problems and greater family needs. Bootstrap results (2000 samples) indicated that these relationships were robust and not sensitive to sampling variability.

The descriptive distributions of the three main variables, including behavioral problems, problematic digital media use, and family needs, were examined, and the results are presented in Table 7. Since normative reference scores for the scales were not available, the term “high level” (elevated level) was operationally defined based on percentile values within the sample distribution. According to the results of the SPSS Explore analysis, the 75th percentile values were 120 for behavioral problems, 36 for problematic digital media use, and 78 for family needs. Participants scoring at or above these thresholds were classified as “high-level.” Approximately one-fourth of the sample fell within these elevated ranges, indicating that a substantial proportion of participants experienced increased levels of problems and needs across all three domains. These findings support Hypotheses H1–H3, which proposed that participants would exhibit elevated levels of problematic digital media use, behavioral problems, and family needs.

Building on the findings, we observed clear support for H3, H4, H5, H6, H8, and H9. H1 and H2 received partial support, indicating patterns consistent with moderate-to-high levels but not uniformly “high” across all comparisons. H7 was also partially supported, with particularly pronounced group differences in behavior problems and family needs.

## 4. Discussion

Beyond statistical significance, the magnitude and structure of our effects warrant careful interpretation. Group differences by SES and parental education were very large (e.g., PMUS-SF η^2^ = 0.86; RBPC η^2^ = 0.73), an uncommon pattern in psychosocial research that may reflect not only substantive inequalities but also measurement features (Likert scaling, potential range restriction) and administration mode. Likewise, inter-subscale correlations within RBPC and FNAT approached unity, and total scores correlated strongly with each other (PMUS–RBPC r ≈ 0.81; PMUS–FNAT r ≈ 0.83), suggesting considerable construct overlap and shared-method variance. Accordingly, we interpret the results as a correlated constellation of family and child risks rather than fully separable drivers, and we refrain from causal claims given the cross-sectional design. Group contrasts by disability add nuance: the ASD subgroup showed higher problematic media use and behavior-problem levels, while multiple disability was associated with the highest family-needs scores; these effects were small-to-moderate (η^2^ ≈ 0.03–0.06) relative to SES/education. Framed within the Family Stress Model and DSMM, the results are consistent with hypotheses that lower SES/education co-occur with greater parent-reported needs and more challenging child behaviors; however, the cross-sectional design and correlated measurement structure preclude directional claims. Future analyses should evaluate whether SES/education attenuate ASD-related differences and whether parental information/support needs mediate associations between context and child outcomes, using multivariable or SEM approaches with appropriate robustness checks.

This study examined levels of children’s problematic digital media use, behavior problems, and family needs in special education contexts, and assessed how these indicators vary by disability type, parental education, and socioeconomic status (SES). The findings indicate high levels among parents and moderate levels among children. Group comparisons show particularly elevated problematic use and behavior problems in the ASD group, alongside graded increases in scale scores at lower education/SES levels (e.g., for the PMUS-SF, *F* = 702.80, *p* < 0.001, η^2^ = 0.86; for the RBPC, *F* = 320.41, *p* < 0.001, η^2^ = 0.73). This pattern aligns with the Family Stress Model, which anticipates more challenging parenting contexts—and thus more adverse child outcomes—in lower-SES settings; it is also compatible with the Differential Susceptibility to Media Effects Model (DSMM), in that media-use indicators appear associated with child characteristics (e.g., ASD) and family context (education/SES). Because the design is cross-sectional, these theoretical arrows indicate hypothesized directions of association; causal inferences cannot be drawn from the present data.

The present findings help situate the heterogeneity surrounding children with special needs in terms of problematic digital media use, behavior problems, and parents’ reported needs. First, when attention is directed to parents’ needs, levels appear elevated. This pattern may be related to gaps in knowledge and skills about how to approach their children, how to support them during leisure time, and how to provide appropriate instructional guidance, as well as to financial constraints that limit the creation of supplementary conditions. It is also consistent with the observation that families with lower educational attainment and socioeconomic status (SES) tend to report greater needs. Accordingly, priority should be given to arrangements that address parents’ educational, financial, and social support needs (Hajizada, 2022; Mumcu, 2023). When needs are examined by disability category, the highest levels are observed among families of children with multiple disabilities. Because “multiple disabilities” refers to the co-occurrence of more than one condition (e.g., ASD with visual impairment, intellectual disability, or physical disability), more complex and elevated needs among these children and their families are to be expected (Browder et al., 2020; Şafak, 2018). Following multiple disabilities, children with ASD constitute the group with the next highest level of family needs and also exhibit the highest levels of both behavior problems and problematic digital media use.

The study indicates that children with special needs obtained mid-range scores on problematic digital media use. When families’ needs are insufficiently met, parents may be hindered in planning effective leisure activities with their children and in developing appropriate instructional strategies (Bent et al., 2025; Seydel et al., 2016). Such limitations may be associated with more prolonged digital media use and, in turn, with higher levels of problematic use among children with special needs. This pattern also underscores gaps in parents’ knowledge and skills for managing and guiding their children’s media use. To provide effective support, it is critical to account for individual differences among children and to offer guidance that helps families foster more regulated and purposeful digital media practices. In this regard, parent education programs and individualized intervention approaches appear to hold promise both for structuring media use and for supporting children’s developmental processes (Girgin & Balcı, 2015; Kaytez et al., 2015). These supports may not only be associated with reductions in problematic media use but also contribute to improvements in the quality of life of families and children. Results further suggest that behavior problems were at mid-range levels. Children with higher levels of problematic digital media use were also observed to exhibit various forms of behavior problems (Davis et al., 2023). Prioritizing the identification and addressing of family needs may therefore be a relevant target for interventions aimed at mitigating these difficulties.

Another finding of the study is the presence of positive, statistically significant associations among problematic digital media use, behavior problems, and family needs. These associations may reflect the dynamics of parent–child interactions during leisure time and the ways in which parents contribute to children’s developmental processes (Roberson et al., 2025). The extent to which family needs are met appears to be linked to parents’ capacity to provide guidance and support in childrearing (Karamipour et al., 2024). In particular, unmet needs related to information access, social support, and education are associated with less effective management of children’s media use and behavior problems. Taken together, these results underscore the relevance of family-focused intervention programs and individualized supports for addressing children’s developmental needs while strengthening parent–child interactions. Developing comprehensive, structured strategies that respond to families’ needs constitutes a promising avenue to target reductions in problematic media use and to enhance children’s behavioral adjustment (Özpolat, 2023).

The findings of the present study are broadly consistent with prior work. Studies by Engelhardt and Mazurek (2014) and Gençtürk (2022) indicate that parental attitudes are closely associated with children’s problematic digital media use. In particular, this literature underscores the importance of parents’ leveraging digital media in ways that support children’s developmental trajectories. Parents’ capacity to guide and scaffold media use is widely viewed as salient for mitigating risks related to media dependence and for fostering healthier media habits. In this context, the current results suggest that structured parent education and guidance programs aimed at strengthening digital media management skills may serve as practical avenues to shape children’s media-use patterns in developmentally supportive directions.

The findings indicate that children’s disability status is associated with variation in parental needs, children’s behavior problems, and digital media use patterns. In particular, parents of children with ASD reported higher levels of needs than parents in other disability groups. This pattern aligns with literature suggesting that children with ASD tend to use digital media more frequently and intensively (Engelhardt & Mazurek, 2014; Mazurek & Wenstrup, 2013). Given the potential positive and negative implications of digital media use for the cognitive, social, and behavioral development of children with ASD, it is important for parents to develop a comprehensive understanding of both the risks and the benefits of these tools. Within this context, structured parent education and guidance services may help families manage digital media use more effectively and tailor strategies to children’s individual needs. Such approaches may not only support more regulated media use but also be associated with strengthened parent–child relationships and more favorable developmental outcomes.

Our findings indicate that parents’ educational attainment and socioeconomic status (SES) are associated with the level of family needs, children’s behavior problems, and problematic digital media use. This pattern is consistent with prior work underscoring the role of parental education and SES in shaping children’s media-use habits and behavioral outcomes (Metin et al., 2017) and documenting meaningful SES-related differences in technology use (Polievková, 2020; Yardi & Bruckman, 2012). Rather than implying that some families are “better” or “worse,” the findings point to the likely influence of differences in institutional access, material/logistical resources, and digital literacy. The large effects observed should be interpreted—without inferring causality—within the structural context of local service infrastructure, school–parent communication channels, and time flexibility. From a policy and practice perspective, improving outcomes in lower-resource groups is more likely to come from reducing access barriers (e.g., appointment schedules, transportation, digital application systems) than from attempting to “correct” parenting behaviors. These results also suggest that variation linked to education and SES likely reflects not only economic resources but also cultural, social, and individual contexts. Accordingly, alongside parent-focused education initiatives, policy and intervention strategies aimed at addressing socioeconomic inequalities may be useful for reducing disparities in these outcomes. Further research that explicitly incorporates cultural context has the potential to provide a more comprehensive understanding of how these associations unfold across settings.

## 5. Limitations and Recommendations

This study relies on a non-probability sample recruited via convenience and snowball methods, with mixed administration (telephone interviews with researcher entry, paper forms transcribed, and online forms), which may introduce interviewer and transcription biases and limit external validity. All focal constructs were measured via the same informant in a single session, raising the possibility of common-method variance and inflating observed associations. Psychometric indices were extremely high (e.g., α ≈ 0.98–0.99) and inter-subscale correlations approached unity, suggesting redundancy that warrants item-level inspection (including ω) and tests of measurement invariance across disability and SES/education groups before making between-group inferences. Multiple group comparisons were conducted without correction; adjusted *p*-values (e.g., Holm/Benjamini–Hochberg), standardized effect sizes (Hedges’ g/Glass’s Δ), and non-parametric robustness checks (e.g., Kruskal–Wallis) are recommended. Analytically, moving beyond bivariate summaries to multivariable regression or SEM would allow joint modeling of SES, education, disability type, child age, and family needs, directly testing Family-Stress/DSMM pathways and attenuation/mediation. For future work, multi-informant designs (parent plus teacher/clinician) and objective media-use indicators (device logs or ecological diaries) would reduce method bias; longitudinal or quasi-experimental evaluations of parent-focused training (digital parenting, service navigation, leisure-time planning) are needed to assess directionality and impact, with pre-registration and fidelity reporting.

This study has several limitations. First, the research data were based on self-reports provided by parents of children with special needs. The accuracy of parents’ reports regarding their children’s behavioral problems and problematic digital media use largely depends on the amount of time they spend with their children and the extent to which they know them well. Consequently, data obtained from parents who spend limited time with their children or have less awareness of their behaviors may be incomplete or biased. To address this methodological limitation, future research could adopt alternative data collection approaches, such as direct behavioral observations or the use of child-appropriate questionnaires and scales designed to capture their own perspectives. Such methodological diversification would enhance the reliability and objectivity of future findings. Moreover, differences in data collection modes may have introduced context-specific measurement factors (e.g., interviewer effects, transcription errors, digital literacy, or perceptions of privacy). For example, online administration may encourage faster completion due to time and location flexibility, whereas telephone interviews might elicit more elaborated responses through real-time interaction with the interviewer. Therefore, descriptive variations across modes may reflect contextual influences rather than true differences in the underlying constructs. Although the present study did not aim to formally test mode effects, process monitoring, transparent reporting, and data quality indicators were employed to ensure sensitivity to this potential source of bias. Additionally, because all variables were collected from a single informant (the parent) in a single session, the high correlations observed among measures may partly reflect common method variance. This suggests that some of the associations may stem from perceptual similarities rather than distinct underlying constructs. Furthermore, measurement invariance (e.g., configural or metric invariance) across groups was not tested. Consequently, group differences reported in this study are based on observed scores, and the assumption that the constructs were measured equivalently across groups remains unverified. Interpretations of group comparisons should therefore be made with caution.

Second, the sample size and demographic diversity were limited. The study included only 357 parents, which substantially constrains the generalizability of the findings to parent populations across Türkiye and internationally. To address this limitation, future research should recruit larger and more demographically diverse samples. In addition, conducting comparative studies that include parents from different geographic regions or countries would not only strengthen external validity but also provide a more comprehensive framework for understanding cultural and regional variation.

Third, this study employed three instruments to assess family needs, behavior problems, and levels of problematic digital media use. Although prior research has reported adequate validity and reliability for these measures, re-testing their performance in the present context across different demographic groups, cultural settings, and larger samples could enhance the generalizability and scope of the findings. In particular, examining how these instruments perform for children of different ages and within varying family dynamics may strengthen the robustness of the results. Additionally, advanced analyses and the development of new theoretical models are needed to clarify potential interactions among variables. For example, investigating the effects of different types of digital media on behavior problems, or the possible mediating or moderating roles between family needs and media use, represents an agenda for more comprehensive future research. Such methodological and theoretical expansion would not only enrich the current knowledge base but also support the development of more meaningful and effective intervention strategies.

The findings clearly indicate the need for more in-depth investigation into the etiology and consequences of problematic digital media use among individuals with special needs. To examine the challenges faced by parents and the scope of their needs more comprehensively, advanced qualitative or mixed-methods studies are warranted. The results suggest heterogeneity in parents’ needs and expectations, with the child’s disability type playing a central role in shaping these patterns. In particular, parents of children with ASD reported higher levels of problematic digital media use and behavior problems relative to other disability groups, which aligns with prior evidence that children with ASD tend to use digital media more frequently and intensively. In this context, developing comprehensive parent education programs and individualized counseling focused on digital media management may be responsive to families’ needs. Such approaches could be associated with healthier digital media habits among children and, potentially, with lower levels of behavior problems (noting that causal inference cannot be drawn from the present cross-sectional design). In addition, longitudinal evaluations of these programs are recommended to assess their longer-term effectiveness and to contribute both methodological and practical insights to the literature.

Experimental and quasi-experimental studies are needed to evaluate the effectiveness of parent-focused programs on leisure-time planning and guidance for children’s digital media use. Such interventions should be rigorously designed and implemented with careful attention to parents’ individual needs and family dynamics. Programs ought to aim not only to facilitate effective and meaningful parent–child communication in digital contexts, but also to build parents’ knowledge and skills for supporting their children. In addition, systematic, multi-method research is warranted to develop a more comprehensive understanding of both the potential benefits and risks of digital media for the developmental trajectories of individuals with special needs. In particular, clarifying the opportunities and challenges that digital media use poses for parents would further advance the knowledge base and inform practice. By detailing the experiences of parents of children with disabilities, the difficulties they encounter, and the potential affordances of digital technologies, this study contributes to the literature and highlights concrete directions for future research and program design.

## 6. Conclusions

In a national convenience/snowball sample of 357 parent–child dyads in Türkiye, we observed elevated parent-reported levels of children’s problematic digital media use and behavior problems, alongside substantial family needs. Patterns varied modestly by disability, with ASD showing higher problematic use and behavior and multiple disability showing the highest family-needs levels. In contrast, gradients by parental education and socioeconomic status were pronounced, consistent with the idea that broader contextual constraints are closely intertwined with everyday media practices and child behavior in special-needs contexts.

The findings indicate that problematic digital media use among children with special needs approaches elevated levels, that their parents report substantial and varied needs, and that children exhibit marked behavior problems. Parents’ needs appear particularly pronounced in the domains of education, financial assistance, and social support. These results highlight the necessity for comprehensive intervention and support mechanisms that not only address the management of children’s behavior problems and problematic digital media use but also enhance parents’ quality of life and overall family functioning. Taken together, the evidence points to the development of systematic and sustainable policies to increase both the accessibility and the effectiveness of educational, economic, and social support services for parents of children with special needs. Importantly, such supports should be structured to enable parents to respond more effectively to their children’s needs while strengthening parents’ psychological and social well-being. Accordingly, individualized approaches and family-centered interventions are positioned to address children’s developmental needs and, in the longer term, to contribute to alleviating the multidimensional challenges faced by parents.

Interpretation requires caution. All variables were measured by one informant in one session; internal consistencies were extremely high and inter-subscale correlations approached unity, indicating construct overlap and shared-method variance. Accordingly, we regard these results as depicting a clustered risk profile rather than isolating independent causal effects. The cross-sectional design likewise precludes directional claims.

Even with these caveats, practical implications emerge. Interventions may focus on: (i) digital-parenting guidance tailored to disability profiles; (ii) information and service-navigation support to help families access community and educational resources; and (iii) structured leisure alternatives that reduce reliance on unsupervised screen time. Given the strong SES/education gradients, equity-oriented targeting is warranted.

Future work should (a) verify measurement invariance across disability and SES/education groups; (b) incorporate multi-informant data (e.g., teacher/clinician reports) and objective media-use indicators; (c) use multivariable/SEM approaches to test pathways posited by the Family Stress Model and DSMM; and (d) pursue longitudinal or (quasi-)experimental evaluations of parent-focused programs with pre-registration and fidelity monitoring. Such designs can clarify mechanisms and inform scalable, family-centered supports.

In this study, comparatively elevated levels of problematic digital media use, behavior problems, and family needs were observed among children with special needs and their families. These indicators were significantly associated with disability type (notably ASD), parental education, and socioeconomic status (SES). A graded pattern emerged whereby all indicators tended to be more adverse at lower education/SES levels. While the findings point to digital parenting education, information/service navigation, and financial/social supports as plausible targets for implementable interventions, no causal inference can be drawn. Future research employing longitudinal or experimental designs should test the directionality and mechanisms of these associations to strengthen translation to policy and practice. In sum, efforts to reduce parents’ social, financial, and educational needs; to promote structured leisure activities for children; and to develop tailored special-education and support programs for diverse disability groups may be associated with healthier digital lives for children with special needs.

## Figures and Tables

**Table 1:** Descriptive statistics for family needs, problematic digital media use, and behavior problem levels.

Scales	Subscales	N	$\bar{x}$	Ss	*α*	ω
Problematic Media Use Scale–Short Form (PMUS-SF)	Problematic Media Use	357	3.19	1.28	0.983	0.99
	Total	357	3.19	1.28	0.983	
Revised Behavior Problem Checklist (RBPC)	Conduct	357	3.05	0.82	0.978	0.98
	Socialized Aggression	357	3.24	0.91	0.975	0.97
	Attention Problems	357	3.11	0.82	0.976	0.98
	Anxiety/Withdrawal	357	3.00	0.87	0.939	0.96
	Psychotic Behavior	357	2.80	0.62	0.825	0.93
	Motor Tension	357	2.42	0.66	0.686	0.83
	Total	357	3.53	1.01	0.993	
Family Needs Assessment Tool (FNAT)	Financial Needs	357	3.05	1.36	0.952	0.96
	Need to Explain to Others	357	3.11	1.78	0.982	0.99
	Informational Needs	357	3.09	1.76	0.987	0.99
	General Support &	357	3.28	1.91	0.991	0.99
	Community Services					
	Total	357	2.87	1.69	0.994	

**Table 2:** Correlations among family needs, problematic digital media use, and behavior problem levels.

	(FNAT) Financial Needs	(FNAT) Need to Explain to Others	(FNAT) Information Needs	(FNAT) General Support & Community Services	(RBPC) Conduct Problems	(RBPC) Socialized Aggression	(RBPC) Attention Problems	(RBPC) Anxiety/Withdrawal	(RBPC) Psychotic Behavior	(RBPC) Motor Tension	RBPC Total	PMUS-SF Total	(FNAT Total
(FNAT) Financial Needs	1	0.905	0.935	0.890	0.836	0.820	0.825	0.822	0.911	0.909	0.848	0.833	0.852
(FNAT) Need to Explain to Others	0.905	1	0.993	0.984	0.744	0.765	0.739	0.724	0.882	0.907	0.773	0.799	0.867
(FNAT) Information Needs	0.935	0.993	1	0.979	0.793	0.809	0.787	0.773	0.906	0.929	0.818	0.812	0.880
(FNAT) General Support & Community Services	0.890	0.984	0.979	1	0.724	0.750	0.719	0.695	0.874	0.909	0.754	0.783	0.858
(RBPC) Conduct Problems	0.836	0.744	0.793	0.724	1	0.985	0.995	0.983	0.934	0.899	0.998	0.792	0.781
(RBPC) Socialized Aggression	0.820	0.765	0.809	0.750	0.985	1	0.980	0.955	0.939	0.881	0.989	0.782	0.787
(RBPC) Attention Problems	0.825	0.739	0.787	0.719	0.995	0.980	1	0.985	0.936	0.897	0.996	0.809	0.774
(RBPC) Anxiety/Withdrawal	0.822	0.724	0.773	0.695	0.983	0.955	0.985	1	0.908	0.889	0.984	0.794	0.761
(RBPC) Psychotic Behavior	0.911	0.882	0.906	0.874	0.934	0.939	0.936	0.908	1	0.929	0.949	0.862	0.858
(RBPC) Motor Tension	0.909	0.907	0.929	0.909	0.899	0.881	0.897	0.889	0.929	1	0.913	0.846	0.859
RBPC Total	0.848	0.773	0.818	0.754	0.998	0.989	0.996	0.984	0.949	0.913	1	0.812	0.798
PMUS-SF Total	0.833	0.799	0.812	0.783	0.792	0.782	0.809	0.794	0.862	0.846	0.812	1	0.827
(FNAT) Total	0.852	0.867	0.880	0.858	0.781	0.787	0.774	0.761	0.858	0.859	0.798	0.827	1

**Table 3:** Family needs, children’s problematic digital media use, and behavior problem levels by child disability status.

Scales	Group	n	$\bar{x}$	F	sd	*p*	η^2^	Post Hoc
Problematic Media Use Scale–Short Form (PMUS-SF)	ID	96	2.90	2.01	1.11	0.077	0.03	
	ASD	162	3.39		1.46			
	MD	31	3.16		0.98			
	VI	16	3.08		1.17			
	HI	20	3.27		0.88			
	OTHER	32	2.99		1.17			
Revised Behavior Problem Checklist (RBPC)	ID	96	3.31	4.30	0.85	0.001	0.06	ASD > ID ASD > OTHER
	ASD	162	3.78		1.16			
	MD	31	3.29		0.75			
	VI	16	3.38		0.66			
	HI	20	3.63		0.80			
	OTHER	32	3.18		0.79			
Family Needs Assessment Tool (FNAT)	ID	96	2.59	2.51	1.55	0.030	0.03	MD > ID
	ASD	162	3.03		1.87			
	MD	31	3.49		1.30			
	VI	16	2.43		1.34			
	HI	20	2.81		1.35			
	OTHER	32	2.62		1.55			

ID: Intellectual Disability, ASD: Autism, MD: Multiple Disability, VI: Visually Impaired, HI: Hearing Impaired OTHER: Learning Disability + ADHD + Physical Disability. Note: (“ASD > ID PMUS: ns, *p* = 0.077”).

**Table 4:** Family needs, children’s problematic digital media use, and behavior problem levels by parental socioeconomic status.

	Group	n	$\bar{x}$	F	sd	*p*	η^2^	Post Hoc
Problematic Media Use Scale–Short Form (PMUS-SF)	1. Low	73	4.44	702.80	0.59	0.001	0.86	1 > 2; 1 > 3; 1 > 4; 2 > 3; 2 > 4; 3 > 4 → 1 > 2 > 3 > 4
	2. Medium	120	4.05		0.45			
	3. High	84	2.60		0.31			
	4. Very High	80	1.36		0.57			
Revised Behavior Problem Checklist (RBPC)	1. Low	73	4.85	320.41	0.23	0.001	0.73	1 > 2; 1 > 3; 1 > 4 2 > 3; 2 > 4 3 > 4
	2. Medium	120	3.88		0.77			
	3. High	84	2.83		0.44			
	4. Very High	80	2.52		0.27			
Family Needs Assessment Tool (FNAT)	1. Low	73	4.53	315.93	0.76	0.001	0.73	1 > 2;1 > 3; 1 > 4 2 > 3; 2 > 4 3 ≈ 4
	2. Medium	120	3.97		1.11			
	3. High	84	1.41		0.73			
	4. Very High	80	1.24		0.73			

Note. (1) 0–20,000 TL, (2) 20,001–50,000 TL, (3) 50,001–200,000 TL, (4) ≥200,001 TL.

**Table 5:** Children’s problematic digital media use, behavior problems, and family needs by parental education level.

	Group	n	$\bar{x}$	F	sd	*p*	η^2^	Post Hoc
Problematic Media Use Scale–Short Form (PMUS-SF)	1. Low	73	4.44	304.49	0.59	0.001	0.63	1 > 2; 1 > 3; 2 > 3
	2. Medium	134	3.80		0.85			
	3. High	150	2.02		0.79			
Revised Behavior Problem Checklist (RBPC)	1. Low	73	4.85	530.39	0.23	0.001	0.75	1 > 2; 1 > 3; 2 > 3
	2. Medium	134	3.85		0.76			
	3. High	150	2.60		0.26			
Family Needs Assessment Tool (FNAT)	1. Low	73	4.53	448.38	1.22	0.001	0.72	1 > 2; 1 > 3; 2 > 3
	2. Medium	134	3.81		0.57			
	3. High	150	1.22		1.69			

**Table 6:** Correlations among Problematic Digital Media Use, Behavioral Problems, and Family Needs (N = 357).

Variables	1	2	3	r (Pearson)	95% BCa Confidence Interval	*p*	Hypothesis
1. RBPC	–	0.812 **	0.798 **	0.812/0.798	[0.771, 0.845]/[0.744, 0.845]	<0.001	H4, H5 Supported
2. PMUS-SF	0.812 **	–	0.827 **	0.827	[0.788, 0.859]	<0.001	H6 Supported
3. FNAT	0.798 **	0.827 **	–	–	–	–	–

Note: *p* < 0.01, two-tailed; bootstrap sample = 2000; BCa = bias-corrected accelerated confidence interval. All correlations are positive and strong in magnitude. The ** symbol indicates statistically significant correlations at the *p* < 0.01 level.

**Table 7:** Percentile Values and “High-Level” Cutoffs for Problematic Digital Media Use, Behavioral Problems, and Family Needs Scales (N = 357).

Scale	25th Percentile (Low)	50th Percentile (Median)	75th Percentile (High Threshold	90th Percentile (Very High)
PMUS-SF	24	34	36	44
FNAT	30	58	78	87
RBPC	54	72	120	123

## Data Availability

The data underlying this study are not publicly available due to ethical/privacy restrictions. De-identified data and analysis materials are available from the corresponding author upon reasonable request and with any required institutional approvals.

## Abbreviations

The following abbreviations are used in this manuscript:

PMUS-SF	Problematic Media Use Scale-Short Form
RBPC	Revised Problem Behaviour Checklist
FNAT	Identifying Family Needs Intermediary
ID	Intellectual Disability
ASD	Autism
MD	Multiple Disability
VI	Visually Impaired
HI	Hearing Impaired
OTHER	Learning Disability+ADHD+ Physical Disability
L	Low
M	Medium
H	High
VH	Very High
ADHD	Attention Deficit and Hyperactivity Disorder

## Appendix A. Kruskal–Wallis Test Results

**Table A1:** Results of the Kruskal–Wallis H Test for Group Differences.

Test	RBPC	PMUS-SF	FNAT
Chi-Square	16.721	15.786	22.657
df	5	5	5
Asymp. Sig. (*p*-value)	0.005	0.007	0.000

The Kruskal–Wallis H test revealed significant differences in behavior problems (H(5) = 16.72, *p* = 0.005), problematic digital media use (H(5) = 15.79, *p* = 0.007), and family needs (H(5) = 22.66, *p* < 0.001) across disability types. The findings in Table A1 indicate that the child’s type of disability is associated with variations in levels of behavioral problems, media use, and family needs. Pairwise post hoc comparisons were subsequently conducted using Mann–Whitney U tests to identify the specific group differences.

**Table A2:** Mann–Whitney U Test Results for Behavior Problems (RBPC), Problematic Digital Media Use (PMUS-SF), and Family Needs (FNAT) by Child’s Disability Type (Holm-Adjusted Post hoc Comparisons, N = 357).

Comparison (Disability Group)	Variable	Mann–Whitney U	Z	*p* (2-Tailed)	*p*_adj (Holm)	Significant?	Direction of Difference (Higher Mean Rank)
1–2	*RBPC*	5820.000	−3.393	0.001	0.015	✅	2 > 1
	*PMUS-SF*	5734.000	−3.583	0.000	0.005	✅	2 > 1
	*FNAT*	5514.000	−3.942	0.000	0.005	✅	2 > 1
1–3	*RBPC*	1098.000	−2.221	0.026	0.19	❌	–
	*PMUS-SF*	1393.000	−0.540	0.589	1.00	❌	–
	*FNAT*	858.000	−3.577	0.000	0.009	✅	3 > 1
1–4	*RBPC*	552.000	−1.813	0.070	0.42	❌	–
	*PMUS-SF*	721.500	−0.393	0.694	1.00	❌	–
	*FNAT*	750.000	−0.152	0.880	1.00	❌	–
1–5	*RBPC*	558.000	−2.964	0.003	0.040	✅ (approaching significance)	1 ≠ 5
	*PMUS-SF*	859.000	−0.750	0.453	1.00	❌	–
	*FNAT*	780.000	−1.330	0.184	0.87	❌	–
1–6	*RBPC*	1452.000	−0.466	0.641	1.00	❌	–
	*PMUS-SF*	1489.500	−0.260	0.795	1.00	❌	–
	*FNAT*	1392.000	−0.803	0.422	1.00	❌	–
2–3	*RBPC*	1208.000	−0.450	0.652	1.00	❌	–
	*PMUS-SF*	991.500	−1.572	0.116	0.63	❌	–
	*FNAT*	969.000	−1.685	0.092	0.53	❌	–
2–4	*RBPC*	1556.500	−0.288	0.774	1.00	❌	–
	*PMUS-SF*	1336.500	−1.294	0.196	0.88	❌	–
	*FNAT*	1432.500	−0.854	0.393	1.00	❌	–
2–5	*RBPC*	2167.000	−1.473	0.141	0.71	❌	–
	*PMUS-SF*	1980.500	−2.137	0.033	0.22	❌	–
	*FNAT*	2040.000	−1.925	0.054	0.35	❌	–
2–6	pdTOPLAM	245.000	−0.088	0.930	1.00	❌	–
	*PMUS-SF*	240.500	−0.181	0.856	1.00	❌	–
	*FNAT*	118.000	−3.040	0.002	0.030	✅	2 > 6
3–4	*RBPC*	270.000	−0.959	0.338	1.00	❌	–
	*PMUS-SF*	284.500	−0.529	0.597	1.00	❌	–
	*FNAT*	237.500	−1.468	0.142	0.72	❌	–
3–5	*RBPC*	378.500	−1.810	0.070	0.42	❌	–
	*PMUS-SF*	471.500	−0.355	0.723	1.00	❌	–
	*FNAT*	322.000	−2.508	0.012	0.11	❌	–
3–6	*RBPC*	136.000	−0.889	0.374	1.00	❌	–
	*PMUS-SF*	157.500	−0.087	0.930	1.00	❌	–
	*FNAT*	111.500	−1.569	0.117	0.61	❌	–
4–5	*RBPC*	196.500	−1.392	0.164	0.78	❌	–
	*PMUS-SF*	242.500	−0.314	0.754	1.00	❌	–
	*FNAT*	223.500	−0.728	0.467	1.00	❌	–
4–6	*RBPC*	207.000	−2.256	0.024	0.18	❌	–
	*PMUS-SF*	288.000	−0.640	0.522	1.00	❌	–
	*FNAT*	275.500	−0.861	0.389	1.00	❌	–
5–6	*RBPC*	207.000	−2.256	0.024	0.18	❌	–
	*PMUS-SF*	288.000	−0.640	0.522	1.00	❌	–
	*FNAT*	275.500	−0.861	0.389	1.00	❌	–

Note. *p*_adj (Holm) = *p*-value adjusted using the Holm–Bonferroni correction. The significance threshold is α = 0.05. Group codes: 1 = Intellectual disability, 2 = Autism, 3 = Multiple disabilities, 4 = Visual impairment, 5 = Hearing impairment, 6 = Other. The findings in Table A2 indicate that, after applying the Holm correction, significant differences remained only in the comparisons where the autism group (Group 2) scored higher than the intellectual disability group (Group 1) across all variables, and where the autism group scored higher than the ‘other’ group on the family needs variable.

**Table A3:** Mann–Whitney U Test Results for Behavioral Problems (RBPC), Problematic Digital Media Use (PMUS-SF), and Family Needs (FNAT) Scores by Monthly Household Income Level (Holm-Adjusted Post hoc Comparisons, N = 357).

Comparison (Economic Income Group)	Variable	Mann–Whitney U	Z	*p* (2-Tailed)	*p*_adj (Holm)	Significant?	Direction of Difference (Higher Mean Rank)
1–2	*RBPC*	1218.000	−8.570	0.000	0.002	✅	1 > 2
	*PMUS-SF*	2537.500	−4.990	0.000	0.003	✅	1 > 2
	*FNAT*	2232.000	−5.796	0.000	0.002	✅	1 > 2
1–3	*RBPC*	56.500	−10.763	0.000	0.002	✅	1 > 3
	*PMUS-SF*	76.000	−11.102	0.000	0.002	✅	1 > 3
	*FNAT*	83.500	−10.695	0.000	0.002	✅	1 > 3
1–4	*RBPC*	3.500	−10.834	0.000	0.002	✅	1 > 4
	*PMUS-SF*	63.000	−10.938	0.000	0.002	✅	1 > 4
	*FNAT*	74.500	−11.000	0.000	0.002	✅	1 > 4
2–3	*RBPC*	523.000	−11.147	0.000	0.002	✅	2 > 3
	*PMUS-SF*	118.500	−12.320	0.000	0.002	✅	2 > 3
	*FNAT*	401.500	−11.301	0.000	0.002	✅	2 > 3
2–4	*RBPC*	341.500	−11.421	0.000	0.002	✅	2 > 4
	*PMUS-SF*	206.500	−11.858	0.000	0.002	✅	2 > 4
	*FNAT*	287.500	−11.570	0.000	0.002	✅	2 > 4
3–4	*RBPC*	919.000	−8.204	0.000	0.002	✅	3 > 4
	*PMUS-SF*	333.000	−10.859	0.000	0.002	✅	3 > 4
	*FNAT*	1035.500	−8.025	0.000	0.002	✅	3 > 4

Note: *p*_adj (Holm) = *p*-values adjusted using the Holm–Bonferroni correction. All differences are statistically significant (*p*_adj < 0.05). Income groups: 1 = Low income, 2 = Middle income, 3 = Upper-middle income, 4 = High income. The findings in Table A3 indicate that, across all variables, scores were higher in the low-income group. As household income increased, scores on behavioral problems, problematic media use, and family needs significantly decreased (p_adj < 0.05). These findings indicate that families with lower income levels experience greater difficulties across all domains.

**Table A4:** Mann–Whitney U Test Results for Behavioral Problems (RBPC), Problematic Digital Media Use (PMUS-SF), and Family Needs (FNAT) by Parental Education Level (Holm-Adjusted Post hoc Comparisons, N = 357).

Comparison (Parental Education Level Group)	Variable	Mann–Whitney U	Z	*p* (2-Tailed)	*p*_adj (Holm)	Significant?	Direction of Difference (Higher Mean Rank)
1–2	*RBPC*	1277.500	−8.995	0.000	0.002	✅	1 > 2
	*PMUS-SF*	2536.500	−5.806	0.000	0.002	✅	1 > 2
	*FNAT*	2308.500	−6.358	0.000	0.002	✅	1 > 2
1–3	*RBPC*	0.500	−12.228	0.000	0.002	✅	1 > 3
	*PMUS-SF*	140.000	−12.197	0.000	0.002	✅	1 > 3
	*FNAT*	81.500	−12.198	0.000	0.002	✅	1 > 3
2–3	*RBPC*	52.500	−14.659	0.000	0.002	✅	2 > 3
	*PMUS-SF*	1679.500	−12.375	0.000	0.002	✅	2 > 3
	*FNAT*	599.000	−13.847	0.000	0.002	✅	2 > 3

Note: *p*_adj (Holm) = *p*-values adjusted using the Holm–Bonferroni correction. All differences are statistically significant (*p*_adj < 0.05). Education groups: 1 = Primary school or below, 2 = High school, 3 = University or above. The findings in Table A4 indicate that, as parental education level increases, scores on behavioral problems, problematic digital media use, and family needs decrease significantly. In particular, parents with a primary school education or below scored significantly higher than all other groups (*p*_adj < 0.05).

Note: “Holm adjustment was applied to pairwise post hoc comparisons within each ANOVA family.” (So it’s clear it’s not across all outcomes.)
